# Antipsychotic quetiapine alters the mouse fecal resistome by impacting antibiotic efflux, cell membrane, and cell wall synthesis genes

**DOI:** 10.1128/spectrum.03804-23

**Published:** 2023-12-15

**Authors:** Yasuhiro Kyono, Jonathan D. Magboo, Elizabeth A. Daley, Stephanie A. Flowers

**Affiliations:** 1 Department of Pharmacy Practice, College of Pharmacy, University of Illinois at Chicago, Chicago, Illinois, USA; University of Guelph College of Biological Science, Guelph, Ontario, Canada

**Keywords:** antimicrobial resistance, hybrid capture, antipsychotics, resistome

## Abstract

The understanding of the antimicrobial activities of non-antibiotic pharmaceuticals is evolving, with growing interest in their implications for antimicrobial resistance (AMR) in gut bacteria. Quetiapine, a frequently prescribed second-generation antipsychotic (SGA), is relevant in this context. While previous studies indicate quetiapine’s role in AMR development *in vitro*, the specific mechanisms and impacts remain undefined. We aim to elucidate quetiapine’s effects on the gut resistome in C57BL/6NHsd male and female mice. Over 12 weeks, mice received quetiapine in their drinking water. We longitudinally analyzed the fecal resistome, contrasting it with a control group receiving regular water. Recognizing AMR genes’ minute presence in metagenomes, we adopted a hybrid capture method to examine AMR genes’ longitudinal trends, correlating them with 16S ribosomal RNA (16S rRNA) sequencing data and validating with qPCR. We also evaluated the minimal inhibitory concentrations for *Escherichia coli* exposed to quetiapine *in vitro* and *Escherichia* species cultured from experimental mouse stool. Quetiapine exposure led to a pronounced increase in the relative abundance of certain AMR gene families within the mouse fecal resistome, notably those linked to antibiotic efflux, phosphoethanolamine transferases, and undecaprenyl pyrophosphate-related proteins. *Escherichia* isolates from quetiapine-exposed mice showed significantly reduced colistin sensitivity compared to isolates from control mice. Our findings mark the first evidence that quetiapine, and potentially other SGAs, may influence AMR evolution in complex *in vivo* microbial communities. This highlights the need for continued investigation into how medications impact the gut resistome, guiding improved clinical practices and antimicrobial stewardship.

**IMPORTANCE:**

This study significantly contributes to our understanding of how certain medications can unintentionally contribute to a major global health issue, i.e., antibiotic resistance. Quetiapine, a widely used antipsychotic medication, was found to increase key resistance mechanisms of gut bacteria to antibiotics in mice. Specifically, these data suggest that quetiapine may target elements of the bacterial cell membrane. If similar effects are found in humans, this medicine could unexpectedly make it harder to treat certain infections. This research emphasizes the importance of being mindful about not just antibiotics themselves, but also about other medications that could inadvertently contribute to this problem. Ultimately, these findings underline the necessity for more in-depth research on the broader impact of pharmaceuticals.

## INTRODUCTION

The gut microbiome of psychiatric patients is uniquely shaped by an interplay of factors, including lifestyle, diet, antibiotic exposure, and hospitalization, which often results in significant differences in microbial phyla characteristics compared to healthy individuals (1, 2). Psychotropic medications are consistently shown to independently impact gut microbiome composition and function; however, the mechanism by which these medications bring changes is not well understood as several factors likely contribute to such modifications (3, 4). An often-neglected variable affecting the microbiome is the potent growth-inhibitory effect of many psychotropic medications on commensal bacteria at gut-relevant concentrations. The antimicrobial properties of antipsychotics have been well documented since 1959, when the antipsychotic chlorpromazine was found to inhibit mycobacterium (5, 6). Since then, large-scale drug screens have identified that second-generation antipsychotics (SGAs), such as quetiapine (QUE), also impact the growth of commensal gut bacteria at estimated physiologic concentrations in the human colon (7). A critical, underexplored question arising from reciprocal drug-microbiome interactions concerns the impact of long-term treatment courses on bacterial evolution within a host.

Patients with severe psychiatric disorders frequently exhibit overlapping risk factors for complex infections. These patients have a high incidence of chronic infection due to patient variables such as increased substance use, challenges navigating the medical system, and socioeconomic factors (8). The described variables further augment interactions with standard antibiotics, often administered as empiric treatment for suspected urinary tract infections, skin and soft tissue infections, and respiratory infections (9). Presently, there is a dearth of recommendations for managing frequently reported infections in psychiatric facilities, particularly for prescribing intensivists who typically lack infectious disease expertise.

Amidst these compounding factors lies the emerging concern of antimicrobial resistance (AMR), which adds another layer of complexity to managing infections in psychiatric patients. Antimicrobial resistance in gut bacteria has been demonstrated to serve as a reservoir of AMR pathogens, which can translocate to other body areas, potentially causing life-threatening illnesses (10). Therefore, gut microbiome-derived AMR bacteria are recognized as an infection risk, even when not directly causing infection. A 2021 study reported that *Escherichia coli* isolated from stool samples of psychiatric patients who are being prescribed psychotropic medications exhibited markedly higher resistance against amoxicillin-clavulanic acid, cephalosporins, meropenem, ciprofloxacin, and tetracycline compared to control volunteers (11). Moreover, the probability of isolating extended-spectrum beta-lactamase-producing Enterobacteriaceae and multi-drug resistant (MDR) organisms was higher in treated psychiatric patients. Current antimicrobial stewardship policies frequently focus on reducing external-source infections; however, understanding patient-specific risk factors for AMR development and dissemination within patients is also of clinical importance.

Transitioning from these clinical observations to a more controlled experimental setting, we sought to emulate the potential impact of quetiapine on the gut microbiota. In our previous investigations, we found that *E. coli* developed MDR after 6 weeks of *in vitro* exposure to quetiapine, even at concentrations that were fourfold lower than those estimated in the human colon, and whole-genome sequencing analysis of quetiapine-exposed isolates revealed mutations in known AMR genes (12). Based on these findings, we hypothesized that chronic exposure of bacteria to SGAs, such as quetiapine, over time can indirectly select for AMR mechanisms in the gut microbial community of patients who often necessitate years of quetiapine treatment. The effect of antipsychotic medication on the gut resistome has not been previously explored.

To build upon our previous findings, in this study, we investigated whether exposure to the SGA quetiapine affects AMR composition in a complex gut microbial community. Quetiapine, a dibenzothiazepine class of SGAs, has experienced a remarkable surge in its use since 2007 for a range of both indicated and off-label applications (13, 14). We exposed C57BL/6NHsd male and female adult mice to quetiapine through drinking water and compared the fecal resistome at the beginning and end of the 12-week treatment course. To enrich AMR genes in the metagenome, which otherwise only constitutes a marginal proportion of the metagenomic reads, we employed resistome capture sequencing to characterize changes in the resistome composition and AMR gene variants. Our initial longitudinal analysis led to the discovery that quetiapine exposure increased the relative abundance of AMR gene families pertaining to antibiotic efflux, the phosphoethanolamine transferases and the undecaprenyl pyrophosphate-related proteins. Consistent with these observations, we found that quetiapine exposure decreased the susceptibility to colistin among *E. coli* cultured from stool samples of quetiapine-exposed mice. This work represents the first major insight into the potential role of SGAs in contributing to AMR development in a complex microbial community.

## MATERIALS AND METHODS

### Test compound and antibiotics

The SGA drug quetiapine fumarate (cat# PHR1856) was manufactured by Sigma-Aldrich (St. Louis, MO, USA). The following antibiotics were purchased from Sigma-Aldrich: ceftriaxone disodium salt hemi(heptahydrate) (cat# C5793), levofloxacin (cat# 28266), and colistin sulfate (cat# PHR1605). Ampicillin sodium (cat# A-301-5) was purchased from Gold Biotechnology (St. Louis, MO, USA).

### Mice

All mouse work was approved by the University of Illinois at Chicago (UIC) Animal Care Committee (#22-063). Adult C57BL/6NHsd male and female mice (6–8 weeks old) were purchased from Envigo (Indianapolis, IN, USA). Prior to the experiment, mice were acclimated to the housing environment for 1 week without disturbance. Mice were housed based on gender and experimental group. Each cage housed three mice with water and food available *ad libitum*. Before the experiment, we weighed mice daily to assess hydration and to establish the baseline water consumption per cage. Mice were randomly assigned to one of the two groups to receive autoclaved tap water alone or water with quetiapine [10 mg/kg of body weight (BW)/day; Sigma]. Assuming 20 of 2,000 captured AMR genes being significantly different, we calculated the required sample size using a conservative estimated dispersion of 0.25 and desired minimum fold changes of 3. With a sample size of nine mice in each group, we calculated a power of 0.8 using an exact test and false discovery rate (FDR) = 0.05. Fresh weighted fecal samples were collected from each mouse individually at baseline and at 12 weeks. Each sample was flash frozen and then stored at −80°C until analysis. During the experiment, a male control mouse died from unknown causes. Since we assessed repeated measures for each mouse, we removed the baseline data for this mouse from our data set.

### Drug administration

We administered a dose of quetiapine to mice at roughly 10 mg/kg of body weight/day, which is approximately equivalent to the maximum prescribed dose of quetiapine in humans at 800 mg/day (15). Direct extrapolation of dosage between animal models and humans is difficult due to significant physiological and metabolic differences between species; however, we selected this dose based on prior behavioral and neurochemical studies in rodents finding appropriate effects within this dose range (16, 17) and to avoid sedative effects observed at higher doses (≥40 mg/kg of BW) (18). We administered quetiapine through the drinking water to avoid stress from daily injection/oral gavage or the surgical implantation of mini pumps, and also to mimic oral administration in human patients. Opaque drinking bottles were used to protect quetiapine from light degradation. To maintain the target drug dose over the course of the experiment, we adjusted the drug concentration in the water every 3–4 days, accounting for the mean daily fluid intake and the mean body weight per cage. Dehydration was routinely monitored over the course of the study using the skin tenting method and by measuring water intake.

### Preparation of pre-capture metagenomic DNA library

We extracted DNA from fecal samples using DNeasy PowerSoil Pro Kits (Qiagen cat# 51804) according to the manufacturer’s instructions. For each sample, we resuspended 1.3 µg of DNA in 130 µL of 10 mM Tris buffer (pH 8.0) and sonicated to 400–500 bp fragments using the Covaris S2 sonicator (Intensity = 4, Duty factor = 10%, and Duration = 55 s). We constructed a fecal metagenomic DNA library (input = 500 ng) using the NEBNext Ultra II DNA Library Prep Kit for Illumina (NEB #E7103) according to the manufacturer’s protocol (version 7.0_9/22) with the following adjustments: (i) during fragment size selection, we used 22.5 µL (Step 3A.2) and 10 µL (Step 3A.6) of the NEBNext Sample Purification Beads; and (ii) at Step 4.1.3, we PCR-amplified libraries for five cycles using the NEBNext Multiplex Oligos for Illumina (96 Unique Dual Index Primer Pairs–Plate 1) (NEB #E6440). We analyzed the fragment size distribution of each library using a 4200 TapeStation System (Agilent Technologies) with the TapeStation D1000 Screen Tape (Agilent Technologies) and confirmed that all libraries have a similar size distribution centered around ~550–600 bp. We quantified the concentration of each pre-capture library using the Qubit dsDNA BR Assay Kit (Invitrogen #32853).

### AMR gene capture sequencing

We pooled seven to eight samples of pre-capture libraries (200 ng each) in 120 µL of 0.1× TE (1 mM Tris-HCl, pH 8.0, 0.1 mM EDTA) and reduced the solution volume to 7 µL using Vacufuge (Eppendorf; ~50 min while heating at 45°C) prior to hybrid capture. We enriched AMR genes according to the standard protocol for myBaits Hybridization Capture for Targeted NGS (Daicel Arbor Biosciences #D10024agAMR; version v.5.02 March 2022) with the following adjustments: (i) we hybridized the pooled libraries at 65°C for 24 hours and used 65°C as the wash temperature; (2) at Step S3.2, we used the KAPA HiFi HotStart system (Roche #7958927001) to PCR-amplify on-bead post-capture libraries for 13 cycles. After PCR, we incubated the samples in a thermocycler (Eppendorf) for 5 min at 95°C to dissociate the PCR products from the beads and purified the supernatant using the DNA Clean and Concentrator-5 Kit (Zymo Research #4014). We quantified the concentration of post-capture libraries using Qubit dsDNA HS Assay Kit (Invitrogen #32851) and prepared an equimolar pool of all libraries for 150-bp paired-end sequencing on an Illumina NextSeq500 platform with 1% PhiX spike-in.

### Negative control sequencing

We attempted to obtain negative control samples for sequencing to monitor contamination at several stages during the construction of our capture libraries. This included: (i) using the Solution S6 from QIAmp PowerFeal Pro DNA kit, which was used to elute fecal DNA, as a template during the preparation of pre-capture metagenomic libraries; and (ii) using 0.1× TE, which was used to pool pre-capture libraries, as a template during hybrid capture and post-capture PCR amplification. For both steps, we failed to obtain a quantity of DNA that was sufficient for sequencing, indicating that there was minimal contamination, if any.

### Analysis of target capture sequencing data

Sequenced reads were analyzed as described by Guitor et al. (19) with a few modifications. Paired sequencing reads were trimmed using skewer (version 0.2.2) (20) and deduplicated using *dedupe.sh* from BBMap (version 38.57) (21). We subsampled reads to 3.16 million reads (i.e., the lowest library size), using the sample command from seqtk (version 1.3; https://github.com/lh3/seqtk.git). Resistance Gene Identifier (RGI version 6.0.1) (22) was used to map trimmed reads against comprehensive antimicrobial resistance database (CARD) reference sequences (version 3.2.5). For mapped gene reads, a filtering process was employed to ensure that only high-quality sequences were retained for further downstream analyses. The filtering was based on three thresholds: mapped read count, the average percent coverage of the gene sequence, and gene reference length, with respective thresholds set at 0.8, 0.8, and 0.8. To prioritize certain filtering criteria, we assigned weights to each threshold (1 for mapped read count, 0.1 for average percent coverage of the gene, and 0.5 for reference gene length). In our data set, we prioritized genes with a high number of mapped reads because this is generally a strong indicator of gene presence and abundance. While the percent coverage of a gene is an important filtering parameter, we have assigned it a relatively lower weight (0.1) to balance our interest in retaining large genes and those with a high number of mapped reads. The rationale behind this is that larger genes, despite being of critical interest, might inherently have lower average percent coverage just due to their size. These weights were used to calculate a weighted score for each gene. Genes were filtered independently for each sample.

Normalization and statistical analysis were performed using the edgeR package in R (23). Normalization of raw gene counts was performed individually for each gene and as an aggregation of gene family counts. Metadata were incorporated to account for group (Control or Quetiapine), mouse ID, and time (baseline or 12 weeks). A generalized linear model with a design repeated measures matrix accounting for within-subject variability was fitted to the data to estimate dispersion and normalize the counts by library size. Genes that were not detected in at least five animals were filtered out, and the model was refitted. Normalized data were log2 transformed, and then the relative change in log-transformed counts (log_2_ fold change, log_2_FC) for each mouse was determined between baseline and 12-week time points. Data points outside two standard deviations of the mean were considered outliers and eliminated. Due to the potential interconnectedness of gene abundance among the same gene family and the possibility of coregulation, the assumption of independence might not hold true. To address this concern, we performed a permutation test with 9,999 iterations for each gene, which is a non-parametric approach that does not rely on the independence assumption. This allowed us to evaluate the association between the response and group variables while accounting for potential interconnectedness. A Levene’s test was employed to detect the differences in the homogeneity of variances between groups. *P*-values from both the permutation tests and Levene’s test were adjusted using the Benjamini-Hochberg method to account for multiple testing. We considered a FDR threshold of 5% (0.05) to be statistically significant.

A principal component analysis (PCA) was performed on quantile normalized data. Euclidean distance matrix was computed using the log-transformed normalized gene count data. Permutational multivariate analysis of variance (PERMANOVA) was conducted using the adonis2 function in the vegan package in R (24) to test for significant differences in gene abundance between the baseline values and the 12-week values for Quetiapine and Control group conditions. Gene mutations were identified using the RGI_*main* function by aligning reads against *in silico* predicted allele variants available in CARD’s Resistome and Variants data (version 4.0.0). All preprocessing and analysis code used for this manuscript can be found at https://github.com/StephanieAFlowers/AAP_AMR_Hybridcapture.

### 16S rRNA sequencing and analysis

We conducted 16S rRNA gene sequencing analysis (V3V4) using the same fecal metagenomic DNA sample as AMR gene capture (primers: forward 5′-tcgtcggcagcgtcagatgtgtataagagacagCCTACGGGNGGCWGCAG-3′ and reverse 5′-gtctcgtgggctcggagatgtgtataagagacagGACTACHVGGGTATCTAATCC-3′; lowercase letters correspond to Illumina Nextera adapter sequences). We sequenced the resulting PCR amplicons on the Illumina MiSeq platform at the sequencing core at Northwestern University. The mothur software (version 1.48.0) (25) pipeline processed the sequencing reads. Each operational taxonomic unit’s (OTU’s) taxonomy was ascertained using the SILVA database (version 132). To evaluate the differences in community compositions between “Control” and “Quetiapine” and “Baseline” groups, we used the analysis of molecular variance (AMOVA). We identified differentially abundant OTUs with the *metastats* function in mothur. Multiple comparison corrections were applied using the Benjamini-Hochberg method, adopting an adjusted *P*-value threshold of 0.05 for significance.

### qPCR metagenomic primer design and analysis

Representative AMR genes of interest were processed against an AMR gene reference file provided by RGI. For each specified gene, its corresponding sequence was extracted from the reference file, and a Bowtie2 (version 2.4.2) alignment index was built for each sample. From these alignment files, consensus sequences were generated using SAMtools (version 1.3.1) with the consensus set to a 75% threshold.

We designed qPCR primers against such consensus sequences while avoiding ambiguous bases within each target gene (primer sequences provided in Table S1). We validated the amplification efficiency for each qPCR primer pair by constructing a standard curve. For each fecal sample, we used 100 ng of extracted DNA as input material and determined the quantity of each target gene against standard curves constructed for each primer pair. We normalized the quantity of each target gene to the quantity of the 16S rRNA gene, which was normalized to the fecal weight from which DNA was extracted.

We analyzed qPCR data by comparing the “Control” and “Quetiapine” groups at 12 weeks. Outliers, defined as values beyond two standard deviations from the mean, were excluded. The data distribution was checked for normality. Statistical comparisons were done using the Mann-Whitney *U* test. A *P*-value below 0.05 was considered significant.

### Isolating *Escherichia* species from stool

We isolated bacteria from frozen fecal samples as described by Ju and Willing (26). Briefly, we added 1 mL of 1× phosphate-buffered saline (PBS) to the tube of frozen samples and incubated it in the 37°C water bath for 5–10 min with occasional vortexing. We homogenized the sample with a wide-pore pipette tip until no large clumps were visible. We made a serial dilution of the fecal suspension in 1× PBS, and for each sample, we plated 100 µL of undiluted, 1/10 and 1/100 diluted solution onto MacConkey agar plates (BD #211387) and incubated them overnight at 37°C. We randomly picked four colonies from each plate and expanded them as liquid culture in Luria-Bertani (Miller) media overnight at 37°C. We conducted a “colony” PCR using 5 µL of the liquid culture as a template to amplify 16S rRNA, which were Sanger sequenced to identify genera, and *ipaH* (invasion plasmid antigen H) (27) regions with the following primers: *16S rRNA*: Fwd- AGAGTTTGATCMTGGCTCAG; Rev-TACGGYTACCTTGTTACGACTT; and *ipaH*: Fwd-CGCTCACATGGAACAATCTC; Rev-AGCTTCCGTACGCTTCAGTAC. All samples yielded PCR products for the 16S rRNA regions, while none yielded the products for the *ipaH* region [except for a positive control DNA from *Shigella flexneri* strain 2457T (ATCC cat# 700930D-5); data not shown).

### Minimal inhibitory concentrations

We evaluated minimal inhibitory concentrations (MICs) for *Escherichia* spp. using the broth microdilution method, as per the Clinical and Laboratory Standards Institute guidelines (28), for ampicillin, ceftriaxone, levofloxacin, and colistin. We incubated bacteria at a final concentration of ∼1.25  ×  10^6^ CFU/mL in growth media containing an appropriate range of concentrations for each tested antibiotic. The final volume in each well was 200 µL, and we performed the assays in triplicate for every strain or isolate for each dilution of antibiotics. After a 24-hour incubation period at 37°C, we defined MICs as the lowest antibiotic concentration that inhibited bacterial growth. For the data analysis, outliers deviating more than two standard deviations from the mean were excluded. We used Wilcoxon rank sum tests to compare log_2_-transformed MIC values.

## RESULTS

### Capture sequencing analysis for mouse resistome after quetiapine exposure

To investigate the *in vivo* effect of quetiapine on fecal resistome, we exposed C57BL/6NHsd adult male and female mice to quetiapine (10 mg/kg of body weight/day) via their drinking water for 12 weeks; mice in the Control group received equivalent drinking water without quetiapine. To maintain the target dose, we adjusted quetiapine concentration in the drinking water every 3–4 days according to their change in body weight and water intake. The presence of quetiapine in the drinking water did not significantly affect either body weight gain (Control—19.7% ± 2.3% vs QUE—18.3% ± 2.1%) or water intake (Control—14.2 ± 0.3 mL/day/cage vs QUE—14.4 ± 0.2 mL/day/cage) throughout the experiment. We collected feces from each animal at the beginning (baseline) and at the end of the 12-week quetiapine treatment and constructed metagenomic DNA libraries from these samples.

To focus our analysis on the dynamics of AMR genes and gene variants due to quetiapine treatment, we enriched AMR genes within the metagenomic libraries using a set of hybrid capture probes designed against reference CARD sequences. This probe set was rigorously designed by Guitor et al. (29) to capture >2,000 nucleotide AMR sequences in clinically relevant bacteria and has been experimentally validated to enrich AMR genes >600-fold in metagenomic DNA isolated from stool. On average, we sequenced 5.1 million pair-end deduplicated reads (range 3.16–6.85 million) and subsampled each library to 3.16 million reads before mapping against the CARD reference sequences. While mapping efficiency ranged from 23% to 55% (717,915–1,748,463 reads), we identified similar numbers of mapped AMR genes in each capture library regardless of the number of mapped reads (Fig. S1), indicating that each library had sufficient read depth for downstream analyses.

### Quetiapine exposure minimally impacts the presence or absence of AMR genes in the mouse fecal resistome

We first analyzed whether quetiapine exposure affected the presence/absence of captured AMR genes. Across 34 capture libraries (eight control and nine quetiapine-treated animals; two time points for each animal), we found 134 unique AMR genes (Table S2). To eliminate data trends driven by a limited number of samples, we only retained genes detected in at least five libraries, resulting in 41 remaining genes. The overall majority (37) of these 41 genes were shared in the resistomes of animals at the beginning and at the end of the 12-week experiment (Fig. S2). One gene, *mexF*, was exclusively present at the beginning of the experiment, whereas three genes (*Erm-43*, *Klebsiella pneumoniae kpnH*, and *mefE*) were exclusively present at 12 weeks. However, we did not find any genes exclusively present in either the Control or Quetiapine-treated group at the 12-week time.

### Quetiapine induces shifts in antibiotic resistance gene families: focus on efflux pump and cell wall/membrane synthesis genes

**Fig 1:**
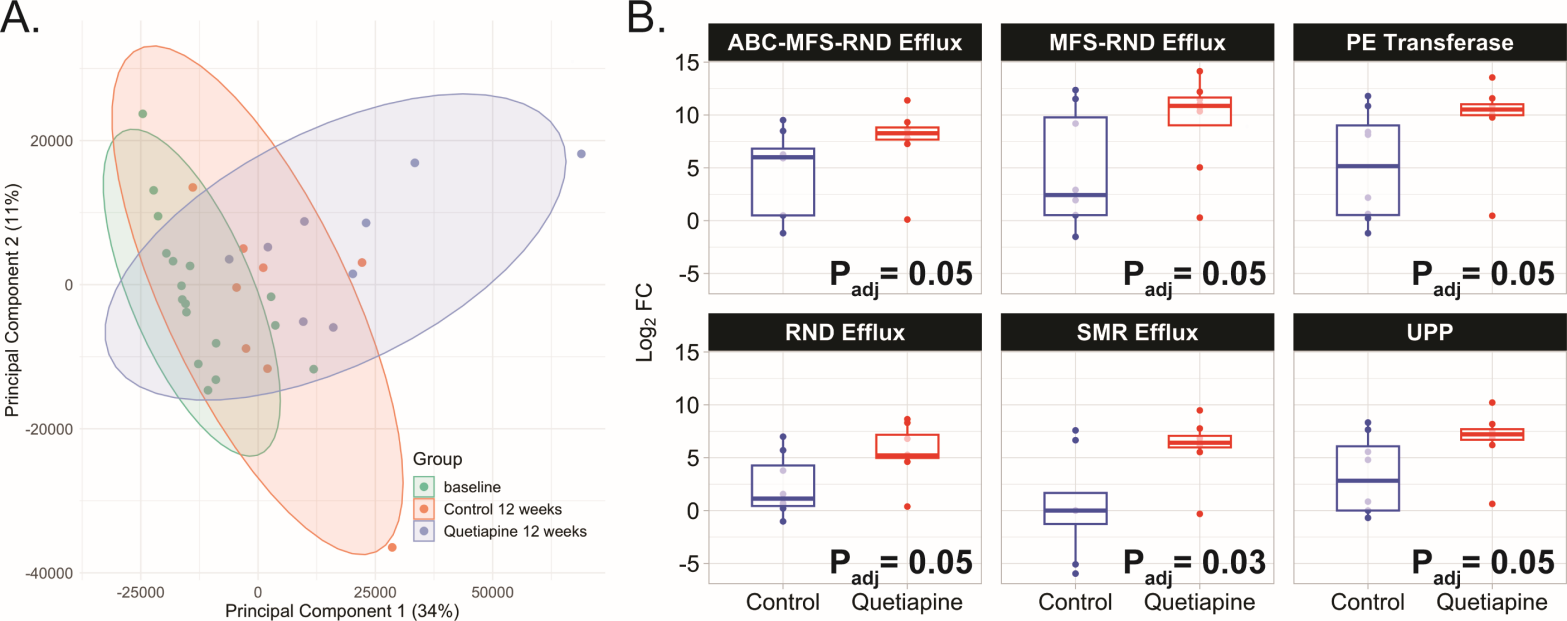
Quetiapine increases the relative abundance of gene families involved in antibiotic efflux, cell membrane, and cell wall synthesis. (**A**) A PCA of antimicrobial resistance gene families shows differential abundance between Baseline and the Quetiapine and Control groups at 12 weeks in the mouse fecal resistomes (PERMANOVA = 0.001). The ellipses represent the 95% confidence interval for each group. (**B**) Log_2_FC of relative gene abundance is visualized between baseline and 12-week libraries of the Control (blue) and the Quetiapine (red) groups for six gene families. Yellow box plots represent the interquartile range. C T0, control at baseline; C T12, control at 12 weeks; Q T0, quetiapine at baseline; and Q T12, quetiapine at 12 weeks.

A PERMANOVA indicated that 12 weeks of quetiapine treatment significantly altered the relative abundance of AMR gene families in the mouse gut resistome (Fig. 1A; PERMANOVA = 0.001). We utilized edgeR to identify specific gene families that are responsible for this effect and found that quetiapine primarily increased the relative abundance of antibiotic efflux resistance mechanisms including: (i) the ATP-binding cassette (ABC) efflux family; (ii) the major facilitator superfamily (MFS) efflux family; (iii) the resistance-nodulation-cell division (RND) efflux family; and (iv) the small multidrug resistance (SMR) efflux family. Quetiapine treatment also increased the relative abundance of AMR gene families associated with cell wall or membrane synthesis mechanisms, specifically the phosphoethanolamine transferases and the undecaprenyl pyrophosphate-related proteins.

Among our data set, the gene *tolC* was uniquely classified by CARD as part of the ABC, MFS, and RND efflux families. TolC encodes a protein that is integral to the outer membrane protein complex in Gram-negative bacteria, and it forms a channel that bridges the outer membrane connecting the periplasmic space to the external environment (30, 31). The relative abundance of *tolC* in the quetiapine group was higher than that of the control [Control log_2_FC from baseline = 4.5 (SD ± 4.02); Quetiapine log_2_FC from baseline = 7.63 (SD ± 3.28); *P*_adj_ *=* 0.05; Fig. 1B]. As an orthogonal validation, we conducted targeted qPCR analysis to measure *tolC* gene abundance in the fecal DNA sample using the consensus sequence based on the capture sequencing data (see Materials and Methods). While the results were directionally consistent with the bait-capture method, the group difference did not reach statistical significance due to high variance (Control—0.102 ± 0.041 vs Quetiapine— 0.284 ± 0.106; *P* = 0.08; Fig. S3).

**Fig 2:**
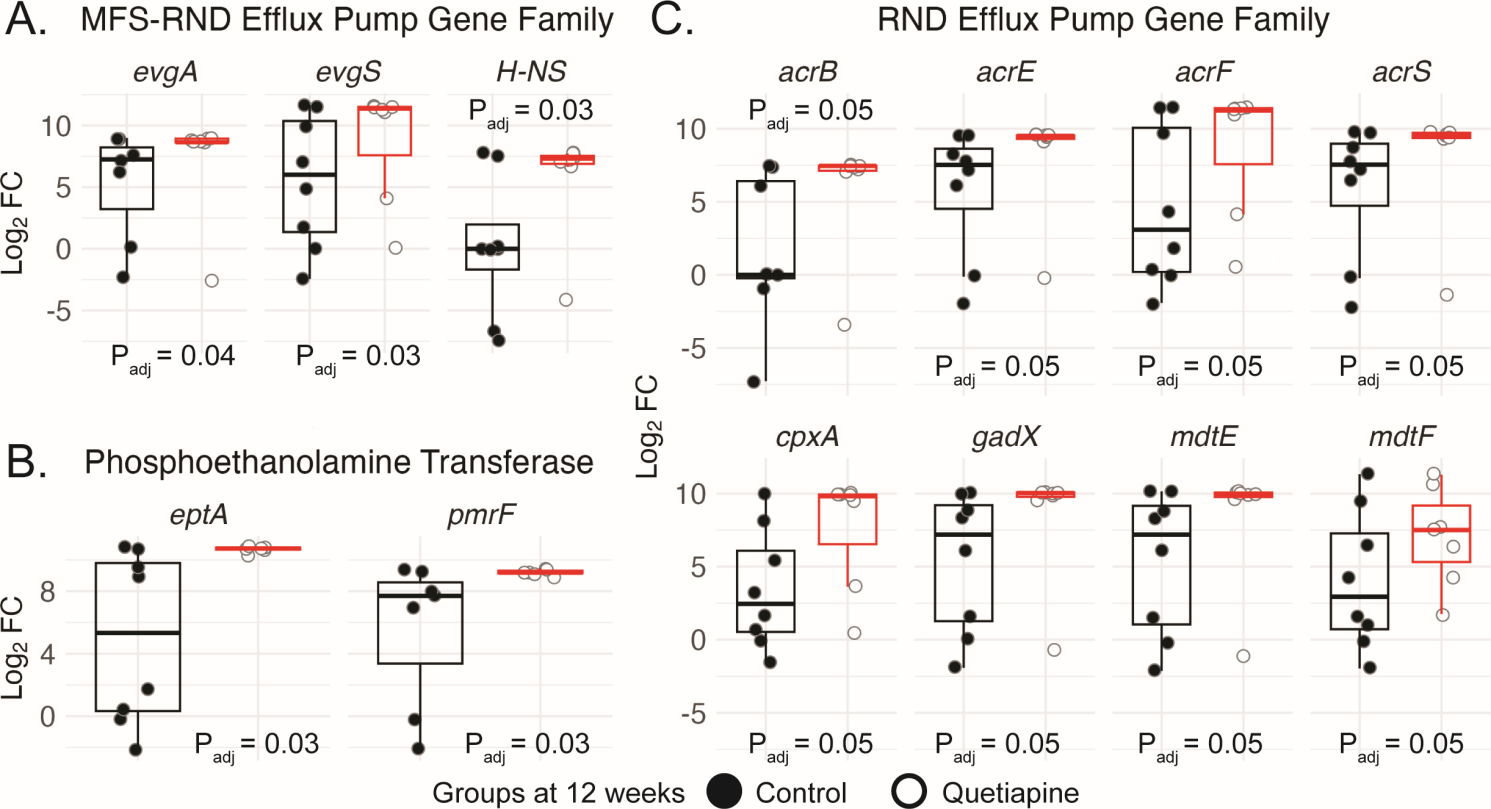
Quetiapine increased the relative abundance of individual AMR genes. Boxplots show log_2_FC of relative gene abundance at 12 weeks between Control (black circles) and Quetiapine (white circles) groups for individual AMR genes that belong to (**A**) MFS and RND efflux family; (**B**) the RND efflux family; and (**C**) the phosphoethanolamine transferases. Black (control) and red (quetiapine) boxplots represent the interquartile range.

Quetiapine exposure also increased the relative abundance of genes that belong to both the MFS and RND efflux families [*evgA*, *evgS*, and *H-NS*; Control log_2_FC from baseline = 4.68 (SD ± 5.48); Quetiapine log_2_FC from baseline = 9.40 (SD ±4.5); *P*_adj_ = 0.05; Fig. 1B]. EvgA and EvgS are two-component regulatory system proteins found in *E. coli* and other enteric bacteria (32, 33). Histone-like nucleoid structuring protein (H-NS) is a DNA-binding protein found in many bacteria that helps to organize the bacterial chromosome and regulate gene expression including those involved in antibacterial efflux (34). The relative abundance of all three genes was respectively higher in the Quetiapine group than that of the control (*evgA—P*_adj_ = 0.04; *evgS—P*_adj_ = 0.03; *H-NS —P*_adj_ = 0.03; Fig. 2A). Targeted qPCR analysis against the *evgA* gene further corroborated our findings (Control—0.070 ± 0.030 vs Quetiapine—0.187 ± 0.070, *P* = 0.02; Fig. S3).

We also detected a significant increase in the RND efflux family after 12 weeks of quetiapine treatment [Control log_2_FC from baseline = 2.30 (SD ± 2.87); Quetiapine log_2_FC from baseline = 5.54 (SD ± 2.58); *P*_adj_ = 0.05, Fig. 1B]. The RND family of efflux pumps plays a crucial role in bacterial resistance to a wide range of antibiotics and other toxic compounds and is one of the most well-studied families of efflux pumps in Gram-negative bacteria (35 – 37). Among the 15 RND genes present in our data set (Fig. S4), several showed significant differences in relative abundance between groups, with adjusted *P*-values as follows: *acrB* (0.05), *acrE* (0.05), *acrF* (0.05), *acrS* (0.05), *cpxA* (0.05), *gadX* (0.05), *mdtE* (0.05), and *mdtF* (0.05; Fig. 2B). Targeted qPCR analysis for the *acrB* gene found a consistent trend (Control—0.090 ± 0.040 vs Quetiapine—0.271 ± 0.113, *P* = 0.08) (Fig. S3).

The *emrE* gene that belongs to the SMR efflux family also showed a greater relative abundance in the quetiapine group [Control log_2_FC from baseline = 0.4 (SD ± 4.81); Quetiapine log_2_FC from baseline = 6.03 (SD ± 2.84); *P*_adj_ = 0.03, Fig. 1B]. SMR efflux pumps are involved in the efflux of a wide range of cationic drugs and other toxic compounds from the bacterial cell (38, 39). Targeted qPCR analysis against the *emrE* gene further corroborated our findings (Control—0.048 ± 0.022 vs Quetiapine—0.128 ± 0.050, *P* = 0.02; Fig. S3).

The second major group of AMR gene families that demonstrated significant changes in the quetiapine-treated group comprises two families crucial for the synthesis and maintenance of the cell wall or cell membrane. The undecaprenyl pyrophosphate-related proteins included only one gene from our data set, *bacA* [Control log_2_FC from baseline = 3.31 (SD ± 3.69); Quetiapine log_2_FC from baseline = 6.76 (SD ± 2.75); *P*_adj_ = 0.05, Fig. 1B]. BacA plays a vital role in the recycling of undecaprenyl pyrophosphate during cell wall biosynthesis, conferring resistance to bacitracin (40). Specifically, BacA is involved in the dephosphorylation of undecaprenyl pyrophosphate (C55-PP) to generate undecaprenyl phosphate (C55-P) (41, 42). C55-P is an essential carrier lipid that can then be utilized in the synthesis of lipid II, which is a precursor to peptidoglycan (42, 43). Consequently, BacA serves as an essential component of the bacterial cell wall biosynthesis machinery. Targeted qPCR analysis for the *bacA* gene found a consistent trend (Control—0.122 ± 0.052 vs Quetiapine—0.379 ± 0.158, *P* = 0.08; Fig. S3).

EptA (also known as PmrC) and PmrF (a.k.a. ArnC) are both phosphoethanolamine transferases that are involved in the modification of lipopolysaccharides (LPS) in Gram-negative bacteria [Control log_2_FC from baseline = 5.13 (SD ± 5.20); Quetiapine log_2_FC from baseline = 9.66 (SD ± 3.90); *P*_adj_ = 0.05, Fig. 1B] (44, 45). Specifically, these enzymes transfer a phosphoethanolamine group to the 4′-phosphate group of the lipid A component of LPS, which reduces the net negative charge of the bacterial outer membrane and can confer resistance to cationic antimicrobial peptides, including polymyxins such as colistin (46). Both *eptA* and *pmrF* showed a higher relative abundance in the Quetiapine group at 12 weeks vs the Control group, each with an adjusted *P*-value of 0.03. Moreover, the Levene’s test results indicated that the phosphoethanolamine transferase-related proteins had a significant difference in variance between the Control and Quetiapine 12-week groups (*eptA P*_adj_ = 7.2 e-07; *prmF P*_adj_ = 1.9 e-03, Fig. 2C). This suggests that the dispersion of this gene family’s abundance might also be affected by quetiapine exposure. Targeted qPCR analysis against the *pmrF* gene further corroborated our findings (Control—0.064 ± 0.030 vs Quetiapine—0.174 ± 0.068, *P* = 0.02; Fig. S3).

### Quetiapine minimally impacts differential detection of AMR gene variants

**Fig 3:**
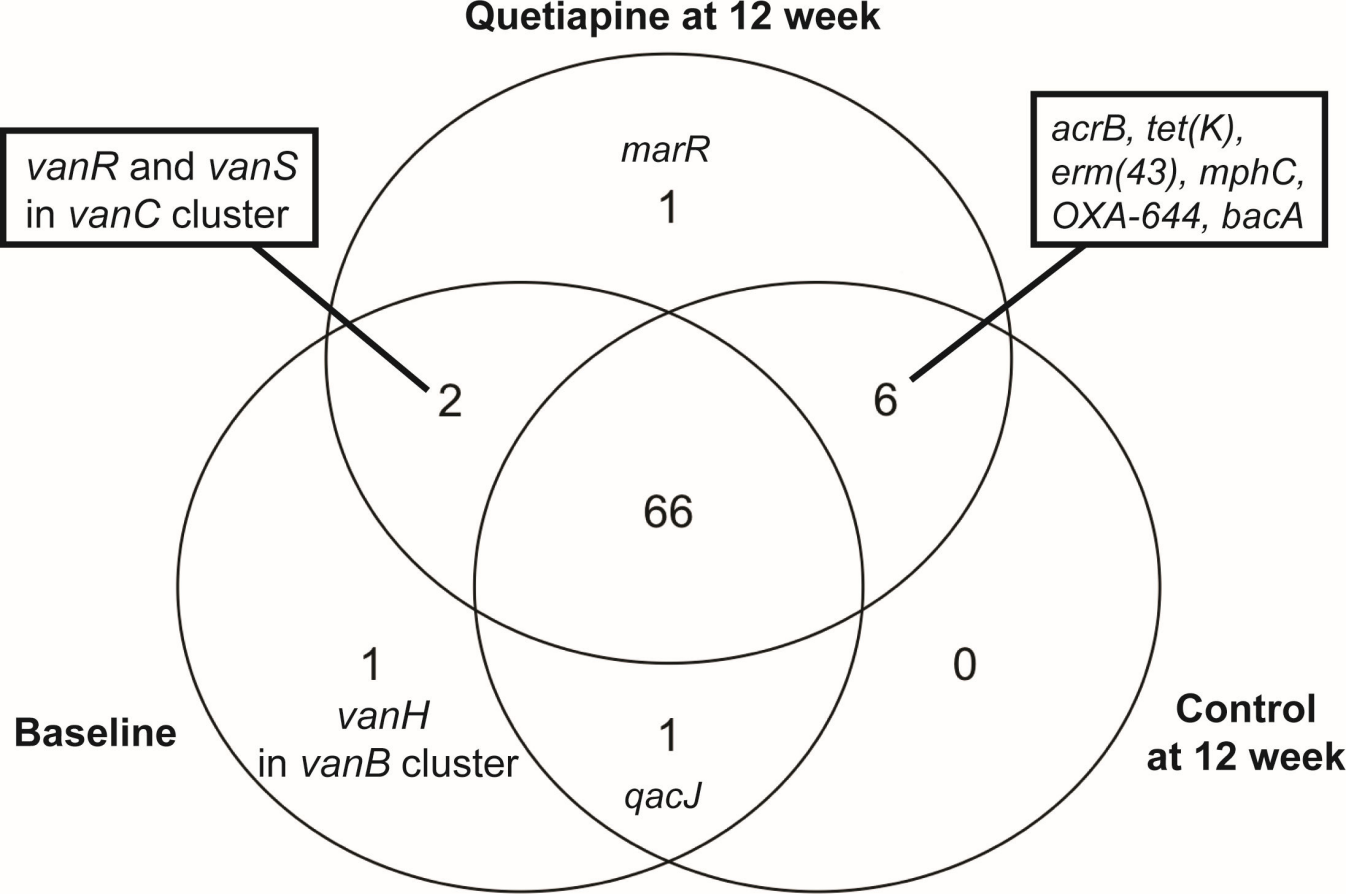
Quetiapine exposure minimally impacts the presence or absence of AMR gene variants in the mouse fecal resistome. The Venn diagram shows the number of captured gene variants shared among the Baseline, Control 12-week, and Quetiapine 12-week conditions.

We used the *main* function in RGI software to detect gene variants in captured AMR genes across three conditions: baseline, control at 12 weeks, and quetiapine at 12 weeks (Fig. 3). To refine our analysis, we included only AMR gene variants observed in a minimum of three animals, which included a total of 77 gene variants in our data set.

The majority (66) of these variants were shared among all three conditions. One variant was unique to the baseline condition (*vanH* gene in *vanB* cluster), and another was unique to the quetiapine at the 12-week condition (*E. coli marR* gene). There were no variants unique to the Control group at 12 weeks. While we found variants in the *marR* gene (Y137H and G103S) in the quetiapine-treated animals from three cages, these variants may not have a functional impact on AMR, as they can be found in the genome of the *E. coli* ATCC 25922, a known antimicrobial susceptible strain (47).

We detected variants in six genes—*acrB*, *bacA*, *Erm(43*), *mphC*, *OXA-644*, and *tet(K*)—that were uniquely present in both 12-week conditions but were absent at the baseline. Because our earlier analysis found that quetiapine treatment increased the relative abundance of *acrB* and *bacA* genes at the 12-week time point, we compared specific variants observed in the Control and Quetiapine at 12-week conditions; however, the same set of variants was present in both conditions. Baseline and Quetiapine at 12 weeks shared variants in *vanR* and *vanS* genes within the *vanC* cluster. Finally, the Baseline and Control at 12 weeks shared a variant in the *qacJ* gene.

### Quetiapine alters gut microbial community structure

Building on our findings concerning the relative abundance of AMR genes, we utilized 16S rRNA amplicon sequencing to assess the microbial community dynamics potentially influenced by quetiapine and to explore whether specific taxa, associated with these AMR genes, showed a parallel increase in abundance.

**Fig 4:**
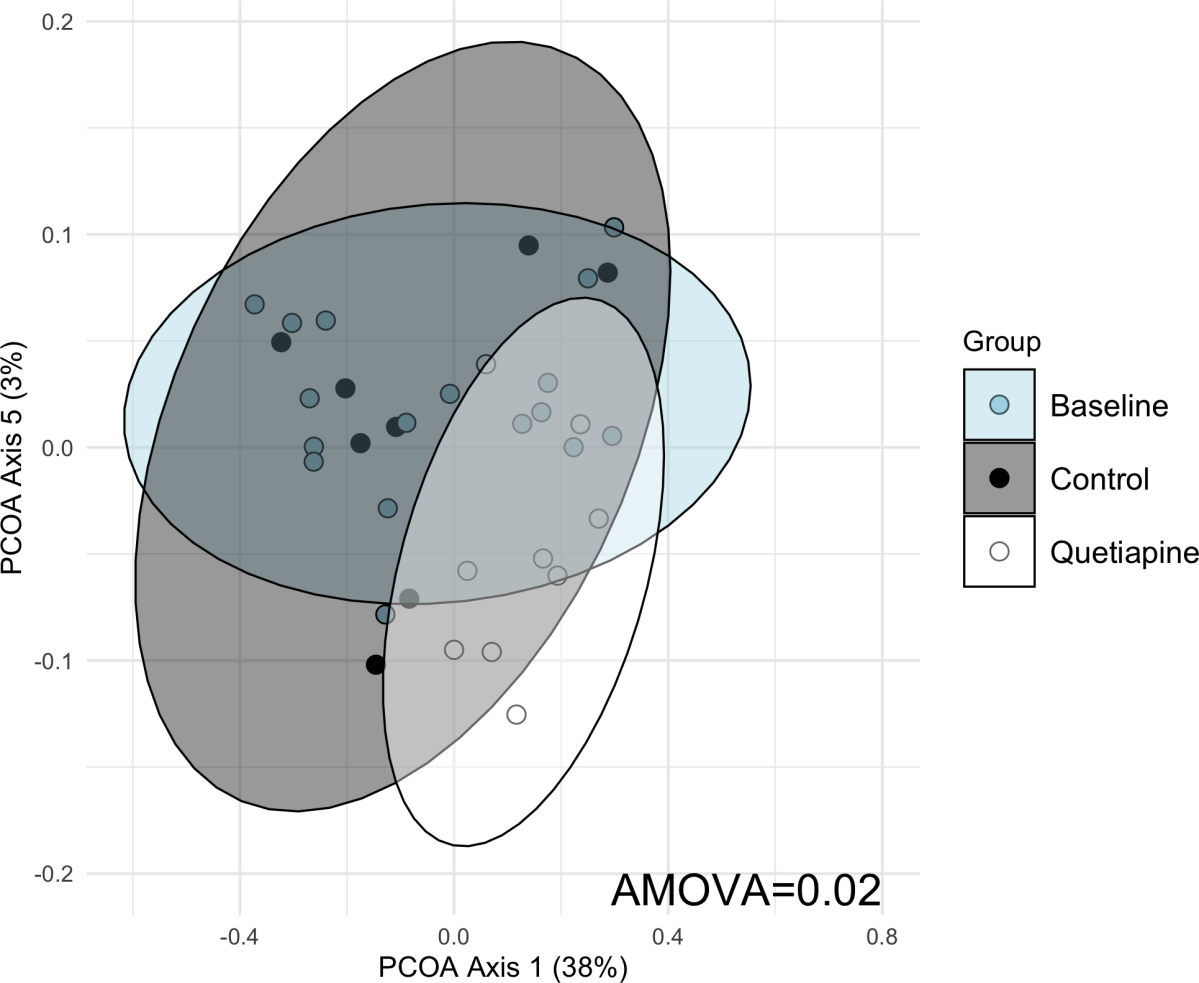
Quetiapine shapes the gut microbiome. Gut community structure in a mouse cohort at baseline and 12 weeks with or without quetiapine exposure. PCoA was used to plot the Yue and Clayton dissimilarity index (AMOVA = 0.02). The ellipses represent the 95% confidence interval for each group.

At 3 months, distinct differences in microbial community structures were observed between the “Control” and “Quetiapine” groups (Fig. 4, AMOVA *P* = 0.02). Among the top 20 identified operational taxonomic units (Table 1), OTU003, classified as *Lactobacillus* (*P*_adj_ = 0.02) and unidentified members of the *Lactobacillaceae* (OTU008; *P*_adj_ = 0.01) and *Lactobacillales* families (OTU012; *P*_adj_ = 0.01), were more prevalent in the Quetiapine group. As the AMR gene families predominant in our Quetiapine group are primarily linked with Gram-negative taxa, the observed increase in Gram-positive species does not align with the shifts in AMR profiles within the gut resistome.

**TABLE 1:** The top 20 most abundant taxa in fecal sample collected at 12-week time point^*a*^

OTU	Phylum	Genera	Control		Quetiapine		*P*_adj_
			Mean (%)	STD	Mean (%)	STD	
1	*Firmicutes*	*Lachnospiraceae* unclassified	32.67	0.06	19.57	0.02	0.14
2	*Bacteroidetes*	*Porphyromonadaceae* unclassified	15.20	0.04	19.85	0.02	0.35
*3	***Firmicutes***	***Lactobacillus***	**8.74**	**0.01**	**20.39**	**0.03**	**0.02**
4	*Bacteroidetes*	*Barnesiella*	3.85	0.01	6.20	0	0.14
5	*Firmicutes*	*Ruminococcaceae* unclassified	3.80	0.01	2.90	0	0.27
6	*Firmicutes*	*Clostridiales* unclassified	3.57	0.01	2.34	0	0.20
7	*Bacteroidetes*	*Alistipes*	2.70	0	4.21	0.01	0.14
*8	***Firmicutes***	***Lactobacillaceae*** **unclassified**	**1.16**	**0**	**2.75**	**0**	**0.01**
9	*Bacteroidetes*	*Prevotellaceae* unclassified	1.99	0.01	2.11	0	0.87
10	*Deferribacteres*	*Mucispirillum*	2.01	0.01	0.90	0	0.20
11	*Bacteroidetes*	*Bacteroidetes* unclassified	1.05	0	1.52	0	0.20
*12	***Firmicutes***	***Lactobacillales*** **unclassified**	**0.68**	**0**	**1.71**	**0**	**0.01**
13	*Bacteroidetes*	*Bacteroides*	2.32	0.01	2.55	0	0.85
14	*Bacteroidetes*	*Bacteroidales* unclassified	0.69	0	1.09	0	0.07
15	*Firmicutes*	*Oscillibacter*	1.02	0	0.96	0	0.87
16	*Firmicutes*	*Lachnospiracea incertae sedis*	1.63	0	0.84	0	0.20
17	*Firmicutes*	*Turicibacter*	6.33	0.02	1.20	0.01	0.14
18	*Firmicutes*	*Firmicutes* unclassified	0.78	0	0.48	0	0.20
19	*Firmicutes*	*Flavonifractor*	0.64	0	0.74	0	0.70
20	*Firmicutes*	*Clostridium XlVb*	0.72	0	0.40	0	0.16

^*a*^ Asterisks denote OTUs that are statistically significant between treatments (*P*_adj_ < 0.05). Bold characters have an adjusted *P* value of under 0.05.

### Quetiapine exposure reduces susceptibility to colistin in *Escherichia* species

**Fig 5:**
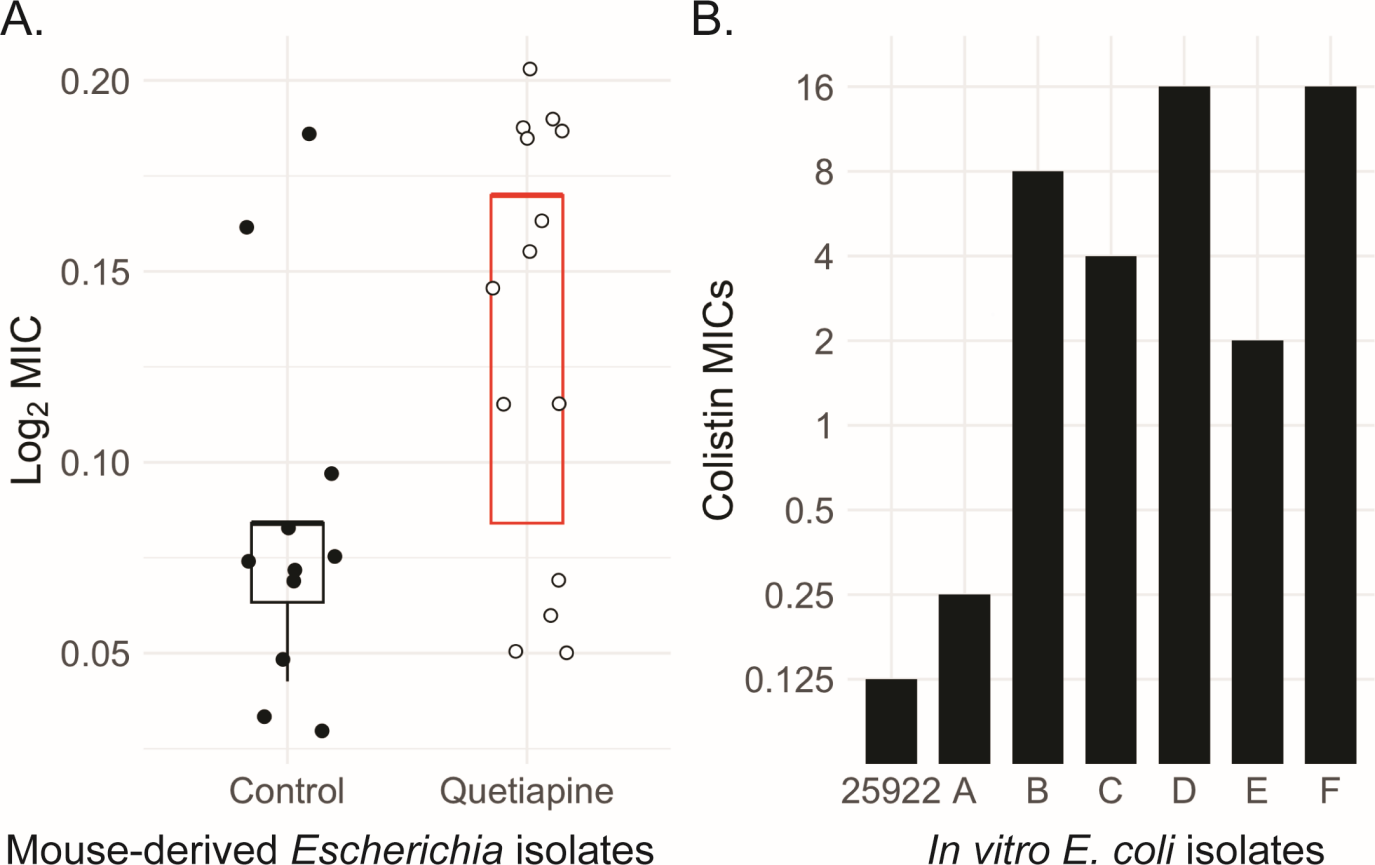
Exposure to quetiapine increases colistin MICs in *Escherichia coli*. (**A**) Colistin MIC for *E. coli* isolates from the mouse feces in Control and Quetiapine groups at week 9. Boxplots with black and white points represent the Control and Quetiapine groups, respectively (Wilcoxon = 0.02). (**B**) Colistin MICs for *E. coli* isolates from the previous *in vitro* experiments (12). ATCC25922 (parent) is a susceptible reference strain. Isolates A–C were derived from three independent cultures of ATCC25922 treated with 10 mg/L quetiapine for 6 weeks, and isolates D–F were derived from three independent cultures of ATCC25922 treated with 100 mg/L quetiapine for 6 weeks.

To investigate the functional implications of these genetic changes, we cultured *Escherichia* spp. from stool samples of mice 9 weeks into the experiment and determined the MICs for several antibiotics (Table 2). Quetiapine-exposed mice showed an increased abundance of PE transferases in the metagenome, prompting us to measure colistin MICs. We found a statistically significant decrease in colistin susceptibility among isolates cultured from quetiapine-treated mice compared to control mice (*P* = 0.02; Wilcoxon rank sum test; Fig. 5A), although clinical colistin resistance was not observed. *Escherichia* isolates cultured from the mouse stool showed no significant changes in susceptibility to ampicillin, ceftriaxone, or levofloxacin (Fig. S5).

**TABLE 2:** Isolates and strains used in this study

ID	Strain background	Relevant properties	Reference or source
Parent WT strain			
ATCC 25922	n/a^*a*^	None	ATCC
*E. coli* derived from *in vitro* quetiapine exposure			
D43LA1 (isolate A)	ATCC 25922	TetR	(12)
D43LB1 (isolate B)	ATCC 25922	TetR, ColR	(12)
D43LC1 (isolate C)	ATCC 25922	TetR, ColR	(12)
D43HA1 (isolate D)	ATCC 25922	AmpR, TetR, ColR	(12)
D43HB1 (isolate E)	ATCC 25922	AmpR, TetR	(12)
D43HC1 (isolate F)	ATCC 25922	AmpR, TetR, ColR	(12)
*E. coli* from C57/6NHsd mouse feces			
C1M3 (A, B, C, D)	Derived from control (cage 1; male #3)	n/a	This study
C2M3 (A, B, C, D)	Derived from control (cage 2; male #3)	n/a	This study
C4M3 (A, B, C, D)	Derived from control (cage 4; female #3)	n/a	This study
Q1M3 (A, B, C, D)	Derived from quetiapine (cage 1; male #3)	n/a	This study
Q2M3 (A, B, C, D)	Derived from quetiapine (cage 2; male #3)	n/a	This study
Q3M3 (A, B, C, D)	Derived from quetiapine (cage 3; female #3)	n/a	This study
Q4M3 (A, B, C, D)	Derived from quetiapine (cage 4; female #3)	n/a	This study

^*a*^ n/a, not applicable.

We wanted to contrast the antimicrobial susceptibility profiles of mouse-derived *Escherichia* isolates with those of our previously published and sequenced *E. coli* isolates, for which we observed that quetiapine exposure (10 and 100 mg/L) for 6 weeks led to AMR development to ampicillin, ceftriaxone, and tetracycline (12). With these same isolates, we measured colistin MICs and observed colistin resistance, particularly in *E. coli* exposed to the higher quetiapine concentration [100 mg/L (isolates D–F); Fig. 5B]. These data suggest that while quetiapine exposure may still subtly influence the susceptibility of gut microbiota to certain antibiotics, the gut microbial environment was protective against the development of AMR.

## DISCUSSION

Mounting evidence suggests that host-targeted drugs with known antimicrobial properties may contribute to the development of AMR in gut microbes, but very little is known about how host drugs affect the resistome *in vivo*. To our knowledge, this is the first study that investigates the effect of quetiapine, a widely prescribed SGA, on AMR dynamics within a gut microbial community. Our findings suggest that quetiapine represents a novel contributor to AMR dynamics in complex microbiota.

Our findings indicated that quetiapine does not significantly influence the presence or absence of unique AMR genes or gene variants in the mouse fecal resistome. The absence of substantial differences between the Control and Quetiapine-treated groups could be attributed to the closed nature of the mouse cohort, in which the animals shared the same environment. A noteworthy observation in most animals was the temporal expansion of the fecal resistome as the study progressed. While consistent across both groups, several factors likely contributed to this observation such as exposure to a novel environment and research staff. The closed nature of this study is a limitation that hinders the detection of unique AMR genes and gene variants. Conducting a similar analysis in a human psychiatric cohort could potentially provide a more comprehensive understanding of the impact of quetiapine on the gut resistome.

While changes were not substantial, we identified several major AMR gene families with significant alterations in relative abundance in response to quetiapine exposure. These gene families are primarily related to antibiotic efflux and cell wall or membrane synthesis. The impact of quetiapine on antibiotic efflux pump families (ABC, MFS, RND, and SMR) could have implications for the efficacy of antibiotics in quetiapine-treated patients, as efflux pumps are known to contribute to multidrug resistance. While functionally distinct, these efflux family genes can be regulated by common transcription factors (48, 49). For example, in *E. coli*, the global regulator MarA activates the expression of multiple efflux pumps (49), including those from the MFS and RND families. Similarly, the AcrAB-TolC efflux pump from the RND family and the EmrAB-TolC efflux pump from the SMR family are regulated by common global regulators such as SoxS and RobA (50 – 53). The overlapping regulation by global regulators suggests that efflux gene expression can be modulated by common environmental or cellular stressors. Efflux pumps are often not selective for specific antibiotics, and this broad substrate specificity could lead to cross-resistance between different classes of antibiotics (37, 54), potentially complicating the antimicrobial treatment of infections in patients.

The observed changes in the phosphoethanolamine transferase family and undecaprenyl pyrophosphate-related protein family, both of which are involved in cell wall or membrane synthesis, may indicate that quetiapine treatment affects bacterial cell wall integrity (40, 45). These findings align with our colistin MICs, which showed that susceptibility to *E. coli* isolates is reduced upon prolonged quetiapine exposure at physiologically relevant concentrations. While it is important to stress that the mouse-derived isolates remained entirely susceptible to colistin, the difference in MICs observed between the Control and Quetiapine groups may have clinical implications. It is also important to note that this effect may be more pronounced in other enteric species that we did not study.

Building on the investigations presented in our preceding publication, we analyzed the colistin MICs of isolates derived from the ATCC 25922 *E. coli* strain that had been subjected to quetiapine in six independent cultures at gut-relevant concentrations for 6 weeks. Our attention was particularly drawn to the isolates demonstrating the highest resistance, specifically isolates D and F. Our previously published whole-genome sequencing of these isolates was notable for mutations in the *acrE/acrR* genes. These mutations may enhance the efflux or reduce the intake of both quetiapine and colistin, rendering the bacteria less “susceptible” to these drugs (55, 56). In contrast, isolate A demonstrated an intriguing deviation. Despite being exposed to quetiapine, it did not develop increased resistance to colistin. Our genomic analysis of this isolate identified mutations in the *arnT* and *pmrB* genes, which are integral to Lipid A modification and resistance to cationic antimicrobial peptides, including colistin (44, 45, 57). This leads us to hypothesize that these mutations may be disruptive, hindering the bacterium’s capacity to modify its Lipid A and maintain its susceptibility to colistin. Further investigations are warranted to understand their role in resistance development.

The intended target for quetiapine is the host’s central nervous system, and it is not known if quetiapine targets the bacterial cell membrane or wall. Pharmacokinetic studies note that about 20% of quetiapine is found in the feces, with only 1% detected in its original form in the urine (58). This is mainly due to the drug’s metabolism into norquetiapine by cytochrome P450 enzyme, CYP3A4. While we are unaware of any bacterial metabolism reports for quetiapine, exploring this could be insightful. There is also the potential for specific *E. coli* strains to bioaccumulate psychotropic drugs, such as duloxetine, impacting their function (59).

Quetiapine is formulated with fumarate, which serves as a counterion to the cationic quetiapine molecule (15). Theoretically, a protonated form of quetiapine could interact with negatively charged components in the bacterial cell wall, such as LPS in Gram-negative bacteria. Whether or not these interactions would significantly impact the bacteria or the drug’s pharmacological action would depend on the strength and specificity of these interactions, which are unknown. Previous research has shown that certain SGAs like quetiapine and olanzapine, as well as the antidepressant sertraline, exhibit activity against *Cryptococcus neoformans*, a pathogenic yeast, with MIC values of 0.5  mg/mL for quetiapine and 0.25  mg/mL for olanzapine (60). While the mechanisms of action for these compounds are not defined, existing literature suggests that such antipsychotic drugs may enhance the permeability of eukaryotic cellular membranes (61, 62).

Our study noted a significant rise in primarily chromosomally encoded Gram-negative AMR genes, which was intriguingly contrasted by an increase in specific Gram-positive taxa. One might posit that certain Gram-negative bacteria, though not increasing in relative abundance, might be intensifying their antimicrobial resistance arsenal, while the observable bloom in Gram-positive taxa could be attributed to other factors. Given that these observed AMR genes are primarily chromosomal, the likelihood of plasmid-mediated transfer is diminished. Although horizontal gene transfer was not encompassed in our study design, the data suggest an expansion of AMR pathways without a proportional rise in the taxa traditionally associated with these resistance mechanisms. This highlights the complexity of microbial dynamics in response to quetiapine and emphasizes the need for a deeper understanding of this interaction.

Although hybrid capture sequencing is a well-established method, primarily known for delineating changes in genetic diversity, its application for investigating AMR in complex microbial communities is a recent advancement (28). While this technique provided us with valuable insights into the potential impacts of quetiapine on AMR, it is important to note that our data largely reflect the relative abundance, rather than direct measures, of specific target sequences within the DNA captured and sequenced. In response to the need for a complementary quantitative approach, we conducted qPCR validation of representative AMR genes. For all targets, qPCR results aligned with the hybridization approach trends, revealing a higher AMR gene abundance in quetiapine-exposed animal feces. However, a proportion of our targets did not achieve statistical significance by qPCR between our experimental groups. While hybrid capture probes can tolerate some sequence variations within target sequences, qPCR primers are highly sensitive to such variations. Therefore, minor sequence variations within primer binding sites, which were otherwise tolerated by the capture approach, resulted in variations in qPCR data. Despite these challenges, our study offers fresh perspectives on how quetiapine might influence AMR in the gut microbiome.

### Conclusions

Our findings underscore the complexity of antibiotic resistance, influenced not only by the direct action of antibiotics but also possibly by exposure to non-antibiotic pharmaceuticals. Characterizing quetiapine’s role in the development of AMR emphasizes the need for further research, comparing diverse medications and their impacts on the gut resistome. To develop population-specific stewardship strategies, it is critical that healthcare providers understand the determinants of a complicated infection, including variables contributing to AMR. Taken together, these findings present a call to scrutinize the potential unintended consequence of pharmaceuticals on antibiotic resistance, a global public health crisis.

## Data Availability

Raw sequencing data and metadata for the hybrid capture experiment and the 16S rRNA amplicon surveys are available at the NCBI Sequence Read Archive (SRA) under accession number PRJNA983335. Raw sequencing data and metadata for the *in vitro E. coli* isolates are available at the NCBI SRA under accession number PRJNA789822.

## SUPPLEMENTAL MATERIAL

Fig. S1 spectrum.03804-23-s0001.tif Captured libraries detected similar numbers of AMR genes.

Fig. S2 spectrum.03804-23-s0002.tif Quetiapine exposure minimally impacts the presence or absence of AMR genes in the mouse fecal resistome.

Fig. S3 spectrum.03804-23-s0003.tif qPCR validation of representative genes that showed an increase in relative abundance using AMR gene capture sequencing.

Fig. S4 spectrum.03804-23-s0004.tif The relative abundance of individual RND efflux family genes.

Fig. S5 spectrum.03804-23-s0005.tif Quetiapine exposure did not alter MICs for ampicillin, ceftriaxone, levofloxacin in *Escherichia* species.

Tables S1-S2 spectrum.03804-23-s0006.xlsx qPCR primer sequences and full list of reported AMR genes.

Supplemental figure text spectrum.03804-23-s0007.docx Legends of supplemental figures and tables.

ASM does not own the copyrights to Supplemental Material that may be linked to, or accessed through, an article. The authors have granted ASM a non-exclusive, world-wide license to publish the Supplemental Material files. Please contact the corresponding author directly for reuse.
