# Supplementary figures and images for: Antipsychotic quetiapine alters the mouse fecal resistome by impacting antibiotic efflux, cell membrane, and cell wall synthesis genes
Source: Microbiol Spectr. 2023 Dec 15;12(1):e03804-23. doi: 10.1128/spectrum.03804-23 (PMC10782992; doi:10.1128/spectrum.03804-23)

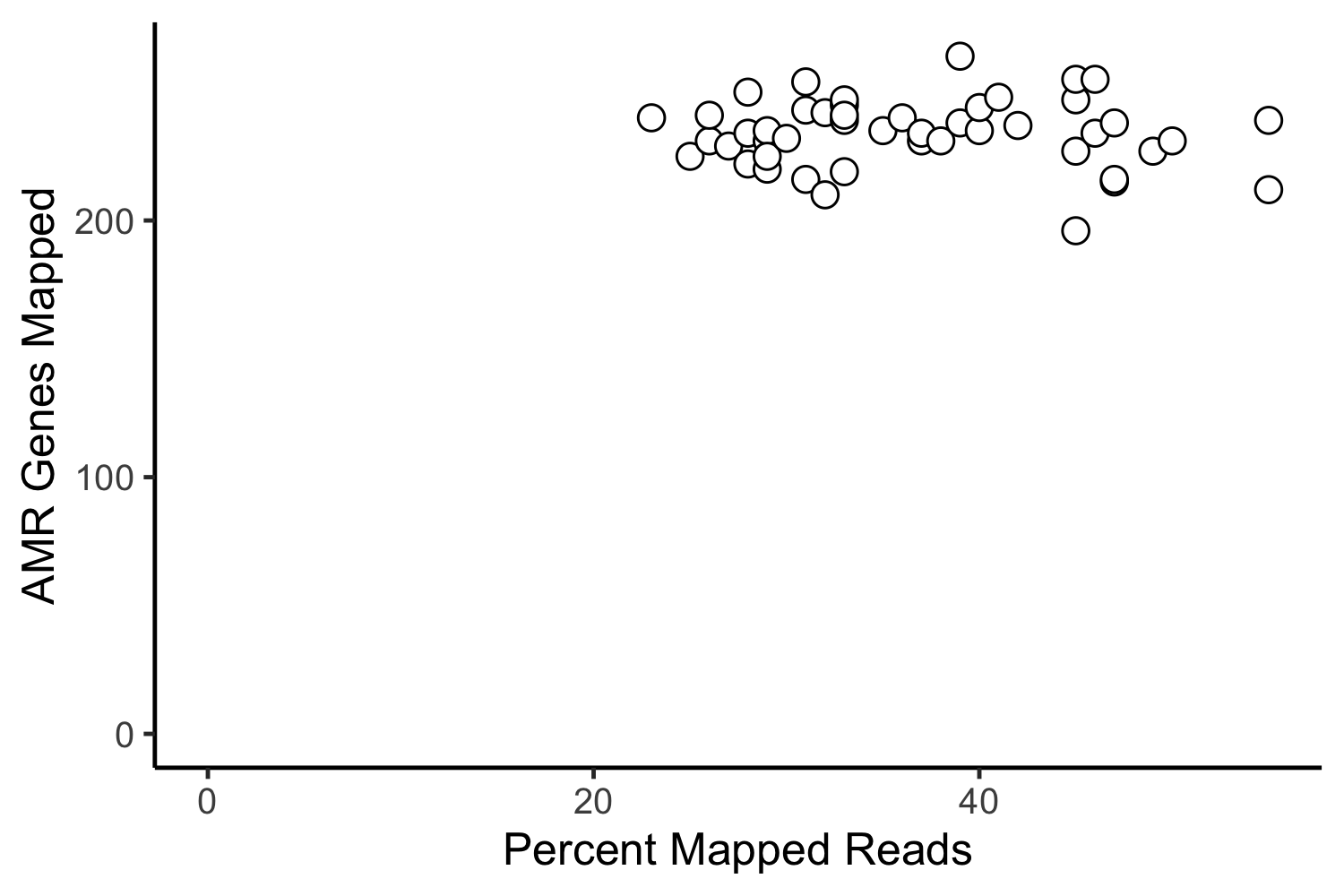

Supplement: Fig. S1 — Captured libraries detected similar numbers of AMR genes. [file spectrum.03804-23-s0001.tif]

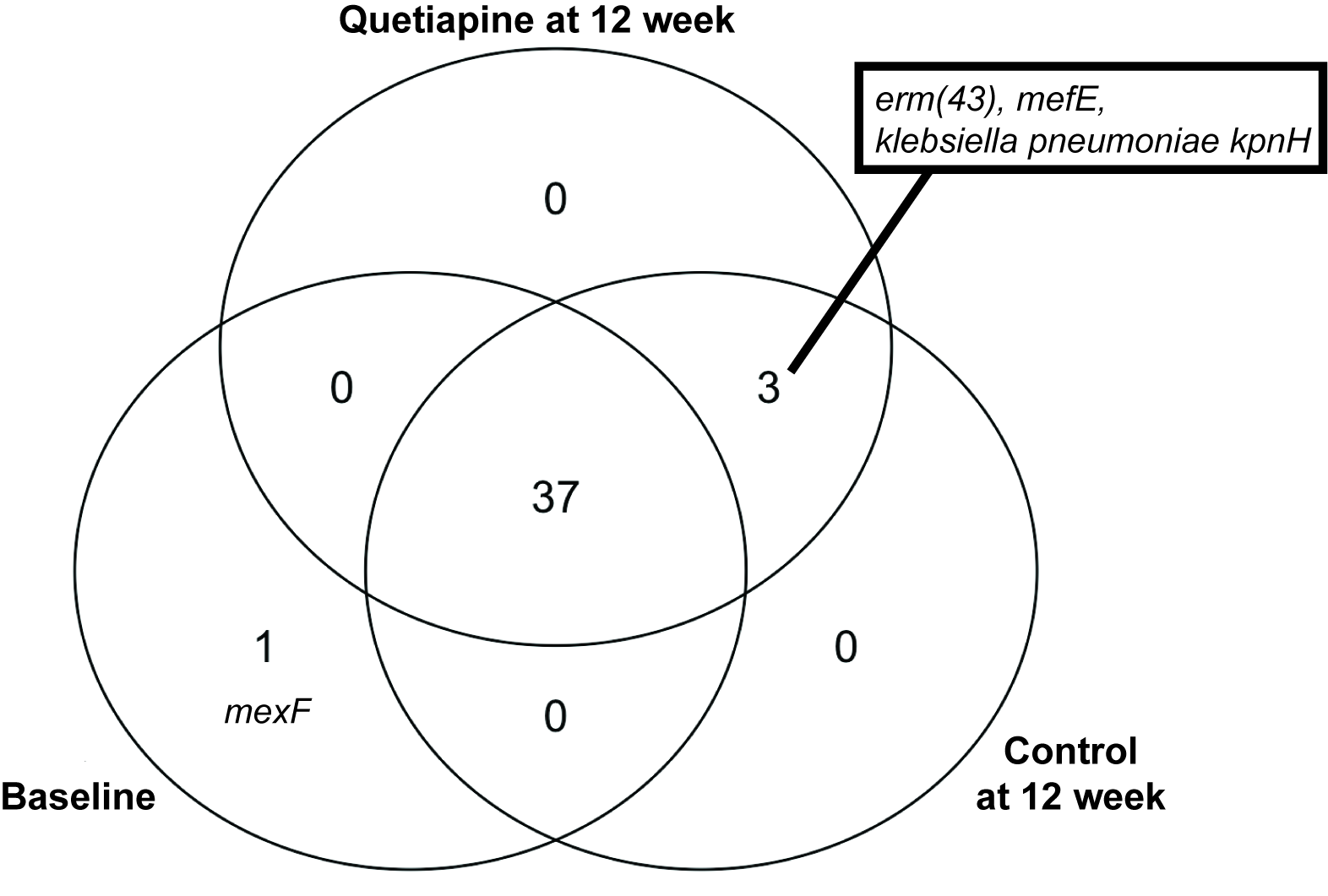

Supplement: Fig. S2 — Quetiapine exposure minimally impacts the presence or absence of AMR genes in the mouse fecal resistome. [file spectrum.03804-23-s0002.tif]

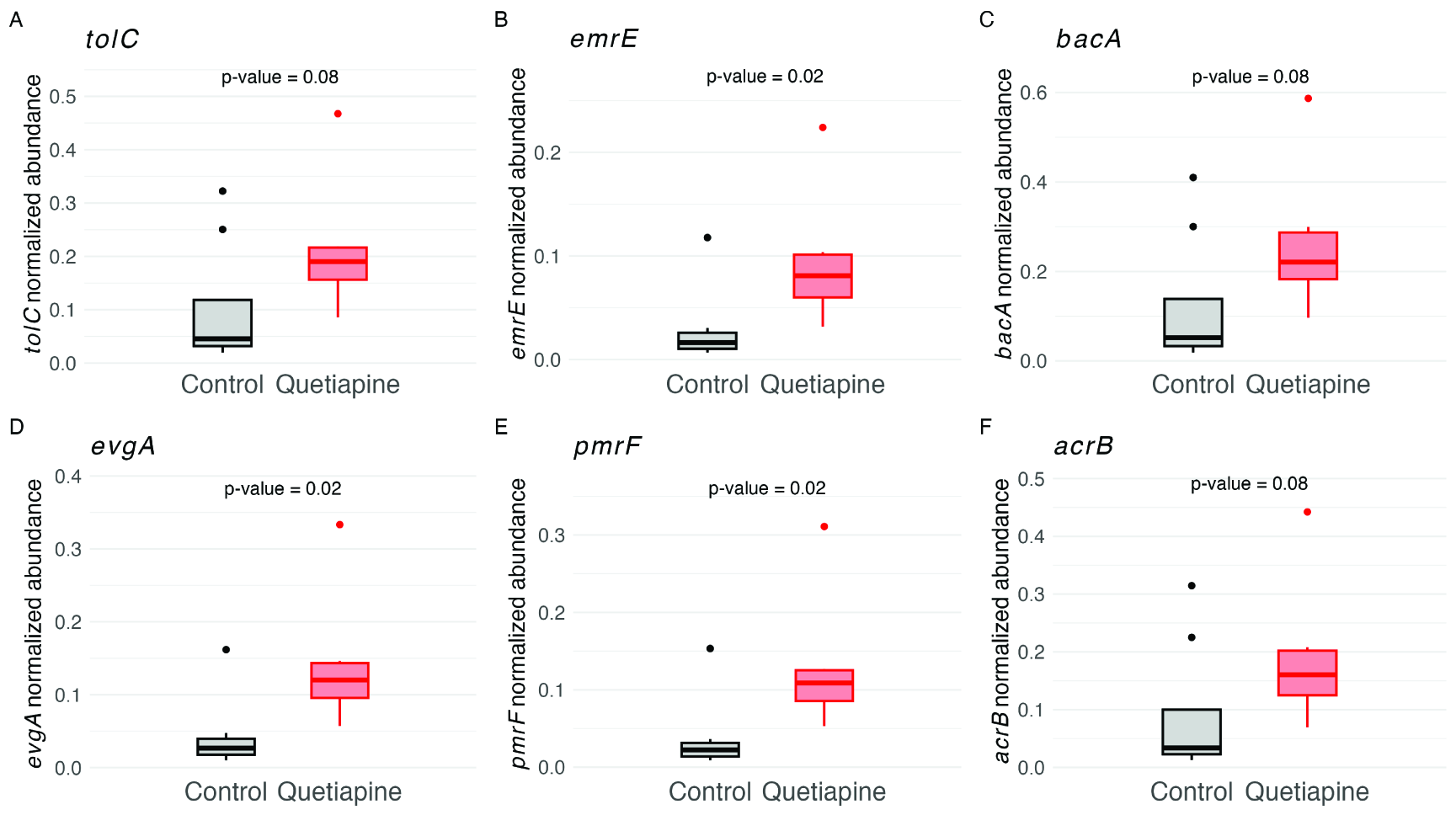

Supplement: Fig. S3 — qPCR validation of representative genes that showed an increase in relative abundance using AMR gene capture sequencing. [file spectrum.03804-23-s0003.tif]

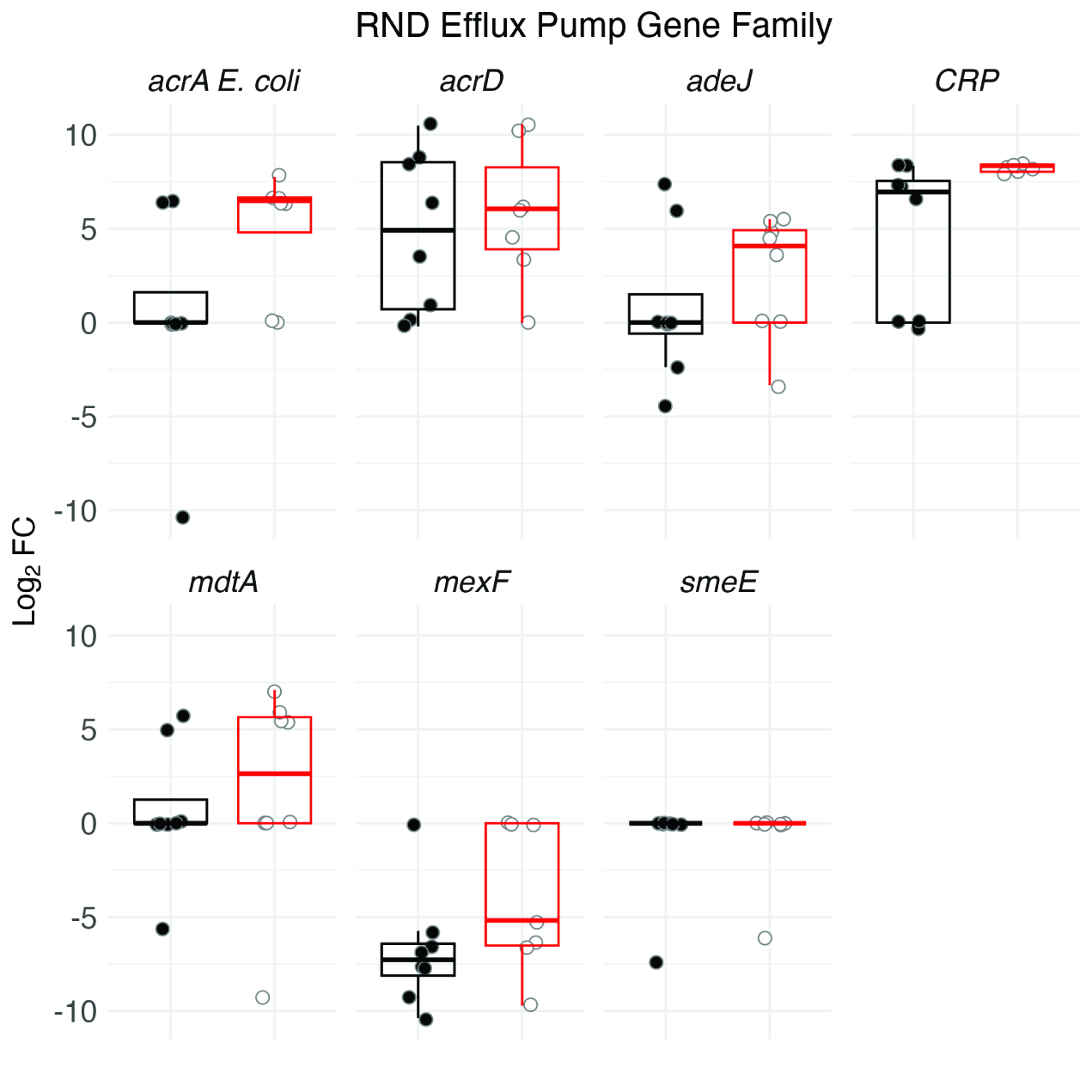

Supplement: Fig. S4 — The relative abundance of individual RND efflux family genes. [file spectrum.03804-23-s0004.tif]

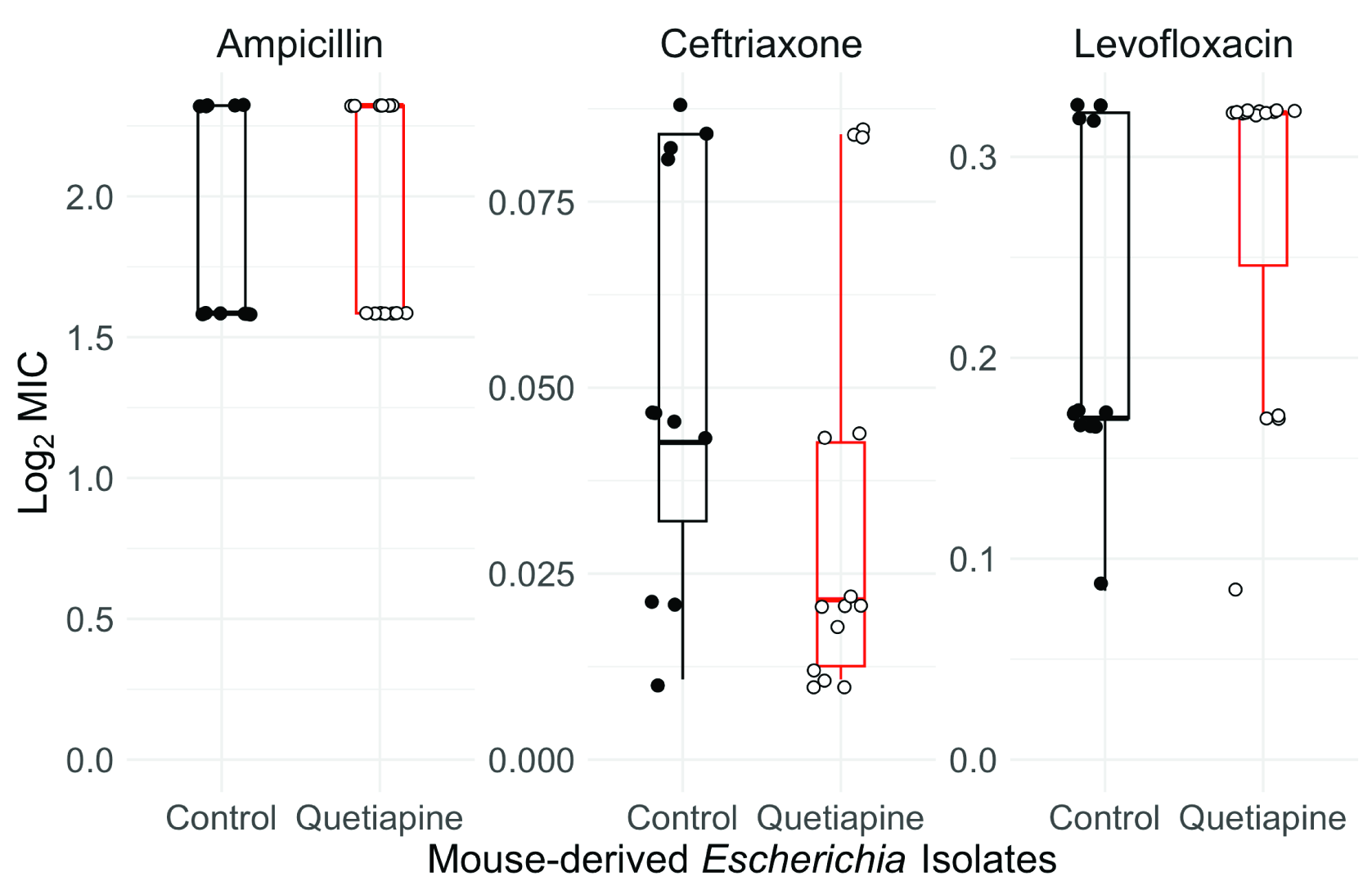

Supplement: Fig. S5 — Quetiapine exposure did not alter MICs for ampicillin, ceftriaxone, levofloxacin in Escherichia species. [file spectrum.03804-23-s0005.tif]
