# Supplementary material for: Antipsychotic quetiapine alters the mouse fecal resistome by impacting antibiotic efflux, cell membrane, and cell wall synthesis genes
Source: Microbiol Spectr. 2023 Dec 15;12(1):e03804-23. doi: 10.1128/spectrum.03804-23 (PMC10782992; doi:10.1128/spectrum.03804-23)
Supplement: Supplemental figure text — Legends of supplemental figures and tables. [file spectrum.03804-23-s0007.docx]

**Supplemental Figure/Table Legends:**

**Supplemental Figure 1**: **Captured Libraries Detected Similar Numbers of AMR Genes**. Before mapping, we subsampled each library to 3.16 million reads (the lowest library size). Each circle represents a unique library. The dot plot shows the % mapped reads against the CARD reference sequences, and the number of unique AMR genes that were detected in each library.

**Supplemental Figure 2: Quetiapine Exposure Minimally Impacts the Presence or Absence of AMR genes in the Mouse Fecal Resistome.** The Venn diagram shows the number of unique captured genes shared among the gut resistome of baseline, Control 12-weeks and Quetiapine 12-weeks conditions.

**Supplemental Figure 3: qPCR validation of representative genes that showed an increase in relative abundance using AMR gene capture sequencing**. We measured quantity of each target using 100 ng of fecal DNA as input material using standard curve method, which was then normalized to the quantity of 16S rRNA.

**Supplemental Figure 4:** **The Relative Abundance of Individual RND Efflux Family Genes.** Boxplots show Log_2_ Fold Change (Log_2_FC) of relative gene abundance at 12 weeks between control (black circles) and quetiapine (white circles) groups for individual RND efflux family genes. Black (control) and red (quetiapine) box plots represent the interquartile range (IQR). None of the genes met criteria for statistical significance.

**Supplemental Figure 5:** **Quetiapine Exposure Did Not Alter Mics for Ampicillin, Ceftriaxone, Levofloxacin** **In *Escherichia* species*.*** MICs for *Escherichia* species isolated from mouse feces at week 9 of the experiment. Boxplots with black and white points represent the Control and Quetiapine groups, respectively (not significant).

**Supplemental Table 1**: qPCR primer sequences used in this study

**Supplemental Table 2**: A comprehensive list of 134 unique AMR genes that were detected by hybrid capture in the fecal samples of the study mouse cohort.
